# Unraveling the genetic basis of oil quality in olives: a comparative transcriptome analysis

**DOI:** 10.3389/fpls.2024.1467102

**Published:** 2024-10-01

**Authors:** AliAkbar Asadi, Elahe Tavakol, Vahid Shariati, Mehdi Hosseini Mazinani

**Affiliations:** ^1^ Department of Plant Molecular Biotechnology, National Institute of Genetic Engineering and Biotechnology (NIGEB), Tehran, Iran; ^2^ Department of Plant Genetics and Production, College of Agriculture, Shiraz University, Shiraz, Iran

**Keywords:** olive, oleic acid, olive oil, oil quality, RNA-Seq, linoleic Acid

## Abstract

**Introduction:**

The balanced fatty acid profile of olive oil not only enhances its stability but also contributes to its positive effects on health, making it a valuable dietary choice. Olive oil's high content of unsaturated fatty acids and low content of saturated fatty acids contribute to its beneficial effects on cardiovascular diseases and cancer. The quantities of these fatty acids in olive oil may fluctuate due to various factors, with genotype being a crucial determinant of the oil's quality.

**Methods:**

This study investigated the genetic basis of oil quality by comparing the transcriptome of two Iranian cultivars with contrasting oil profiles: Mari, known for its high oleic acid content, and Shengeh, characterized by high linoleic acid at Jaén index four.

**Results and discussion:**

Gas chromatography confirmed a significant difference in fatty acid composition between the two cultivars. Mari exhibited significantly higher oleic acid content (78.48%) compared to Shengeh (48.05%), while linoleic acid content was significantly lower in Mari (4.76%) than in Shengeh (26.69%). Using RNA sequencing at Jaén index four, we analyzed genes involved in fatty acid biosynthesis. Differential expression analysis identified 2775 genes showing statistically significant differences between the cultivars. Investigating these genes across nine fundamental pathways involved in oil quality led to the identification of 25 effective genes. Further analysis revealed 78 transcription factors and 95 transcription binding sites involved in oil quality, with BPC6 and RGA emerging as unique factors. This research provides a comprehensive understanding of the genetic and molecular mechanisms underlying oil quality in olive cultivars. The findings have practical implications for olive breeders and producers, potentially streamlining cultivar selection processes and contributing to the production of high-quality olive oil.

## 1 Introduction

The well-balanced composition of fatty acids in olive oil enhances its stability against oxidation, making it a valuable addition to a healthy diet. The high level of unsaturated fatty acids and the low level of saturated fatty acids in olive oil have made it have positive effects on cardiovascular diseases and cancers (Yubero-Serrano et al., 2019; Jimenez-Lopez et al., 2020). Oleic acid, which makes up 55-83% of the total fatty acid content, is the main fatty acid found in olive oil, while other fatty acids present include linoleic acid (2.50-21.00%), palmitic acid (7.5-20.00%), stearic acid (0.5-5.00%), and linolenic acid (<1%) (International Olive Council (IOC), 2019). Olive oils with high levels of oleic acid and low levels of linoleic, linolenic, and palmitic acids are regarded as suitable oil quality and have superior nutritional (Salimonti et al., 2020). The quantities of these fatty acids in olive oil vary depending on genotype, environment, and their interactions (Mele et al., 2018; Navas-López et al., 2020). Furthermore, the cultivar genotype plays a crucial role in determining the quality of the olive oil (Sánchez de Medina et al., 2015; Comlekcioglu et al., 2022).

Iran is renowned in the Middle East as one of the regions with the greatest diversity of olive germplasm (Hosseini-Mazinani et al., 2014). Previous research has been limited on the transcriptome-level evaluation of Iranian olive oil quality, focusing on factors such as ripening stage and harvesting time (Parvini et al., 2014; Zaringhalami et al., 2015; Razeghi Jahroomi et al., 2016; Fahadi Hoveizeh et al., 2023), cultivation location effects (Asheri et al., 2015; Taslimpour et al., 2016; Amiri-Nowdijeh et al., 2018), and environmental temperature impacts (Fahadi Hoveizeh et al., 2023; Karamatlou et al., 2023). In an investigation conducted by Parvini et al. (2015), quantitative real-time PCR was employed to analyze the expression patterns of two key genes: stearoyl [acyl-carrier-protein] 9-desaturase (*OeSAD*) and omega-6 fatty acid desaturase (*OeFAD*); throughout the stages of fruit development. The results revealed elevated levels of *OeSAD* expression and decreased levels of *OeFAD2‐2* expression in Mari and Koroneiki cultivars, leading to an increased ratio of oleic to linoleic acid. In another study conducted by Razeghi-Jahromi et al. (2021), 195 differentially expressed transcripts involved in various fruit developmental stages were detected using cDNA-AFLP in the Mari and Shengeh cultivars. Hence, investigations on Iranian olive cultivars to specify genes influencing oil quality have not been performed into the olive transcriptome utilizing advanced sequencing methods such as RNA-seq. On the other hand, several studies have been conducted in the world regarding olive transcriptome to compare cultivars based on the compositions and biosynthesis of fatty acids and metabolites at different developmental stages (Xiaoxia et al., 2020; Liu et al., 2021; Rao et al., 2021; Niu et al., 2022). In a study conducted by Hu et al. (2023), two cultivars with different fatty acid compositions were examined. It was stated that fatty acyl-ACP thioesterase B, SAD, and FAD2 genes are important in determining the composition of fatty acids. A study was done by Asadi et al. (2023) aiming to perform a meta-analysis of olive RNA-seq data, identify 41 key genes involved in fatty acid biosynthesis, and assess the significance of them (Asadi et al., 2023). The study highlighted beta-ketoacyl-[acp] synthase II (FabF), SAD, and FAD2 as crucial genes influencing the oleic acid levels. In a subsequent study by Unver et al. (2017) aimed at sequencing wild olives, RNA-seq was utilized to identify gene expression patterns related to oil biosynthesis. The research revealed a correlation between decreased FAD2 expression and increased SAD expression with the accumulation of high levels of oleic acid in olives. In a study done by Li et al. (2015), the overexpression of SAD genes in transgenic potatoes led to an increase in linoleic acid levels in membrane lipids, leading to enhanced cold acclimation. In another study, the OeFAD genes were found to desaturate linoleic acid, resulting in the production of C18:3 fatty acids and contributing to the cold acclimation of olive trees (D’Angeli et al., 2016). In many studies, different transcription factors, such as WRKY, MYB, Dof, bZIP, Hox, RGA, LEC, and WRI1, have been identified as effective in the biosynthesis of fatty acids (Manan et al., 2017a; Kong et al., 2020; Yan et al., 2021; Yang et al., 2022). The WRI1 transcription factor has been extensively studied for its central role in regulating plant fatty acid biosynthesis and oil biosynthesis in plants (Kong et al., 2019, 2020).

The objective of this article is to conduct an RNA-seq analysis on *Olea europaea* cv Mari and *Olea europaea* cv Shengeh as two Iranian olive cultivars, for the first time, aiming to uncover the genetic and molecular mechanisms that influence oil quality. Additionally, the study seeks to identify differentially expressed genes and pathways linked to oil quality. Due to this factor, Mari and Shengeh were selected as the two most extreme Iranian olive cultivars for their oil quality, as indicated by prior research (Hosseini-Mazinani et al., 2013; Parvini et al., 2015). Mari (deposition number IRCul-02 in Iranian Olive Catalog) and Shengeh (deposition number IRCul-13 in Iranian Olive Catalog) cultivars have been examined by Hosseini Mezinani from 2013 (Hosseini-Mazinani et al., 2013) in different aspects. Morphological characteristics of both cultivars were analyzed for fruit, stone and leaf based on UPOV-IOC (The International Union for the Protection of New Varieties of Plants-International Olive Council) parameters (Hosseini-Mazinani et al., 2013). Molecular characterization of the studied cultivars was also analyzed using SSR and chloroplast markers (Hosseini-Mazinani et al., 2014). Previous studies have determined that the Mari had higher oleic acid content and an overall more favorable fatty acid profile compared to Shengeh (Parvini et al., 2015, 2016; Amiri-Nowdijeh et al., 2018; Razeghi-Jahromi et al., 2021, 2022). Therefore, Mari was selected for its superior oil quality due to its high oleic acid and low linoleic acid content, whereas Shengeh was chosen for its lower oil quality because of its low oleic acid and high linoleic acid content. This research endeavors to provide valuable insights into the genetic basis of these cultivars, potentially leading to the development of targeted breeding programs and the improvement of olive cultivation practices.

## 2 Materials and methods

### 2.1 Plant materials

Based on the previous studies, Mari and Shengeh were selected as two extreme Iranian olive cultivars (*Olea europaea*) in terms of oil quality. Mari and Shengeh are two reference cultivars in Iran that are preserved in Tarom Olive Germplasm Collection (Tarom Olive Research Station, Tarom city, Zanjan province) which represented one of the most important olive germplasm collections in Iran. Mari and Shengeh cultivars have a medium vigor and sparse canopy with erect habit of growth. All studied trees were 22 years old grown by drip irrigation and were planted in North–South-oriented rows at a 5 × 6 m planting pattern. Tarom is the main region in terms of the area under olive tree cultivation with latitude of the north 36°47’, the longitude of the east 49°6’ and the altitude of 369 m. Based on previous studies (Parvini et al., 2014, 2015), the biosynthesis of fatty acids in Mari and Shengeh cultivars reaches its maximum value in 150 DAF, and after this time, the process of fatty acid biosynthesis will be stable. For this reason, fruit sampling of the Mari and Shengeh cultivars was done at Jaén index four (around 150 DAF). In each cultivar, three trees were selected as replicates and 20 fruits were randomly chosen around the canopy (at a distance of around two meters from the ground) and the samples were immediately frozen in liquid nitrogen and stored at -80^°^C for further analyses.

### 2.2 Oil extraction and fatty acid analysis

In each cultivar, three trees were selected as replicates, and 10 fruits were randomly chosen around the canopy from each replicate for GC analyses. Oil was extracted from the mesocarp tissues of 10 fruits from Mari and Shengeh cultivars by cold pressing method. The extracted oil was transferred into a dark glass and then stored in dark place at 4°C. The fatty acid composition of the oil samples was determined by GC as FAMEs according to European Regulations (EEC 2568/91) and the results were expressed as a percentage of the total. FAMEs were recovered by shaking off a solution of 0.1 g oil and 1mL of n‐Hexan with 0.2mL of 2N methanolic potassium hydroxide. FAMEs analyses were carried out on a ACME 6100 Younglin Capillary Gas chromatograph equipped with a FID (VICI, Valco, Houston, TX, USA), using a fused‐silica capillary column (60m×0.32mm×0.5mm film thickness, Teknokroma, Barcelona, Spain). The injector, detector and oven temperatures were 240, 250, and 185^°^C, respectively. Helium was used as the carrier gas with a lineal flux of 1mLmin^-1^ and a split ratio of 1:50. The experiment was down in triplicate and the mean fatty acids of the cultivars were compared by t-test and the difference between the means was investigated at the probability level of 95%.

### 2.3 RNA-seq analysis

Total RNA was extracted from olive mesocarp tissue of 10 fruits in each replicate using RNeasy Plant mini kit Qiagen (QIAGEN, CA, USA, Cat. Number: 74904) according to the manufacturer’s instructions. DNA was removed by treatment with RNAase-free DNAase at 37°C for 30 min. The quality of extracted RNAs was checked by 1% agarose gel electrophoresis, measuring the RNA concentration, and A260/230 and A260/280 ratios by Nano Drop spectrophotometer. Finally, the RNA extracted from each replication was pooled in equal proportions to create the final RNA sample for each cultivar. This sample was then sequenced using Illumina HiSeq 2500 in paired-end layout with a length of 150 nucleotides. Quality control of raw RNA-seq reads (in FASTQ format) was performed using FastQC (v0.11.8) (http://www.bioinformatics.babraham.ac.uk/projects/fastqc/) while discarding adaptors, ambiguous nucleotides, and low-quality (<20) were trimmed by Trimmomatic (v0.32), excluding reads with final length less than 50 bases. The high quality cleaned reads were aligned onto the Farga (*Olea europaea*) (https://www.ncbi.nlm.nih.gov/assembly/GCA_902713445.1) genome as the reference by means of the Hisat2. The read counts were calculated using the HTSeq to estimate the count of uniquely mapped reads for each of the samples. Finally, the read count matrix was applied for differential expression analysis. The differential expression analysis was carried out by edgeR and false discovery rate (FDR) <0.01 was used to find the DEGs. Finally, DEGs with log-fold change ≥ 2 were selected for downstream analysis.

### 2.4 Pathways enrichment analysis

The identified DEGs was used for pathway enrichment analysis by local pipeline and in-house scripts. The identified biosynthetic pathways were divided into four groups according to the fatty acid biosynthesis pathway and factors affecting oil quality. Important paths were determined according to the results of our previous study (Asadi et al., 2023) in each group and the role of effective genes in them was investigated.

### 2.5 Transcription factors identification

Identification of TFs effective in oil quality was identified by PlantTFDB website (v5.0) (http://planttfdb.gao-lab.org/index.php) (Jin et al., 2014; Tian et al., 2019). For this purpose, the protein sequences of differentially expressed genes were used. In this method, the protein sequences are compared with the protein sequence of TFs in *Arabidopsis thaliana* as model, and the TFs that get the best hit were selected. Also, in order to identify the TFs located upstream of the identified key genes in the RNA-seq analysis, TF enrichment analysis was used on the PlantTFDB website and significant TFs were determined along with the target genes.

The protein-protein interactions of up and down-regulated genes and identified TFs were determined by the STRING (v12.0) (https://string-db.org/) (Szklarczyk et al., 2021, 2023) and plotted using Cytoscape (v3.10.1) (https://cytoscape.org/index.html) (Shannon et al., 2003).

## 3 Results

### 3.1 Determining the fatty acid profile

**Figure 1:**
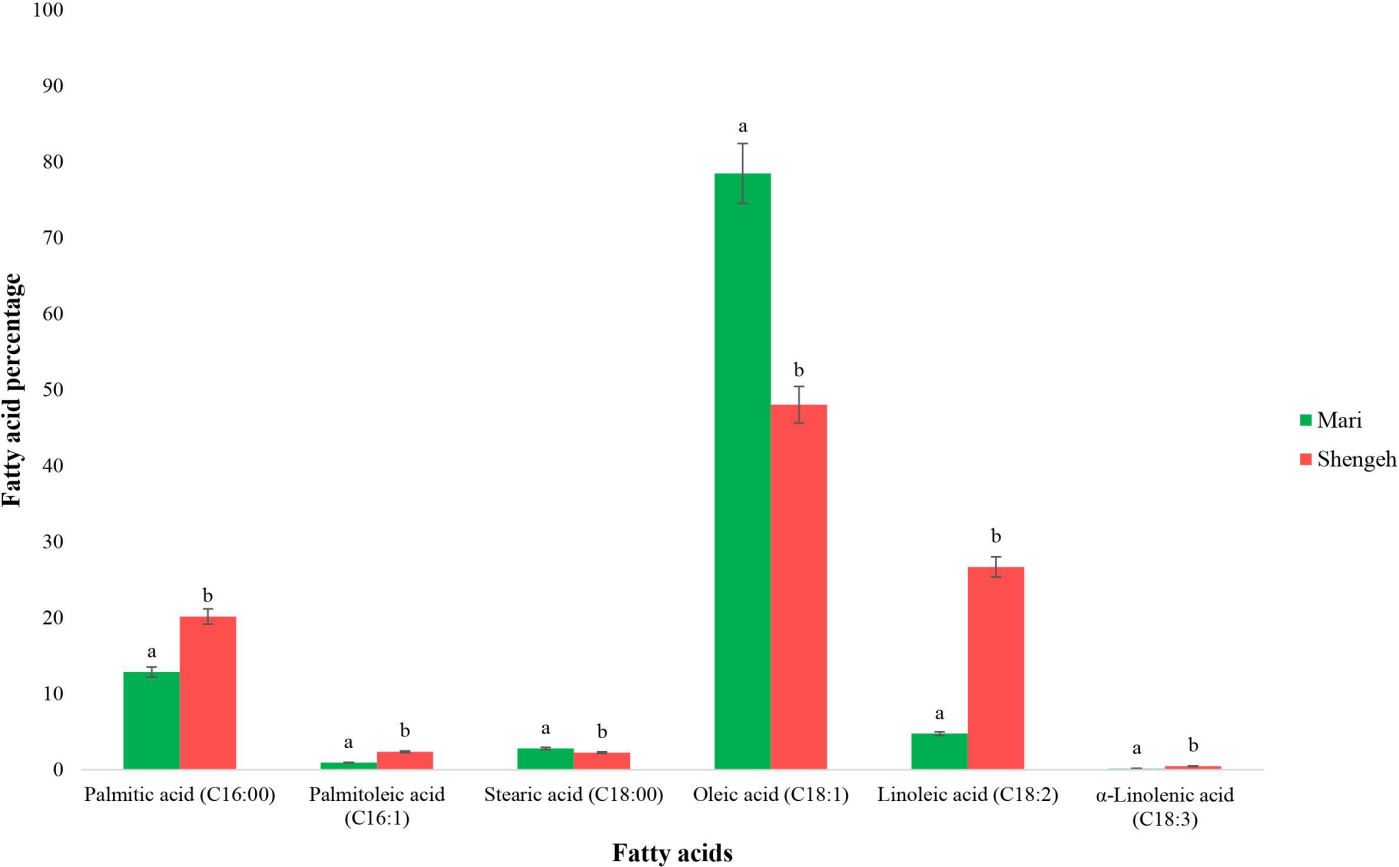
Fatty acid compositions of Mari and Shengeh cultivars at Jaén index four (around 150 DAF). The green hue represents Mari’s fatty acid profile status, while the red hue signifies Shengeh’s status. Statistical comparisons were made between the two studied cultivars for each of the fatty acids separately, and means that do not share a letter are significantly different at 95%.

The GC analysis was performed to determine the fatty acid profiles of the two selected cultivars at Jaén index four (around 150 days after flowering (DAF)). The GC results showed that the main fatty acids in olive oil in both cultivars were palmitic acid (16:00), palmitoleic acid (16:1), stearic acid (18:0), oleic acid (18:1), linoleic acid (18:2), and alpha-linolenic acid (18:3) (**Figure 1**). The results indicated that palmitic acid was dominant saturated fatty acid and oleic acid was dominant unsaturated fatty acid in both selected cultivars. Also, the mean comparison of two cultivars shows a significant difference at the 95% probability level in each of the investigated fatty acids (**Figure 1**). The results showed that the percentage of oleic acid in Mari’s mesocarp (78.48%) was significantly higher than in Shengeh (48.04%), and the percentage of linoleic acid in Mari (4.76%) was significantly lower than in Shengeh (26.69%). Based on the percentage of oleic acid and linoleic acid, the oleic acid to linoleic acid (O/L) ratio in Mari was 16.49% and 1.80% in Shengeh. Moreover, the study findings indicated that the ratio of total unsaturated fatty acids to total saturated fatty acids is a key factor affecting the oxidative stability of olive oil. The ratio of total unsaturated fatty acids to total saturated fatty acids in Mari (5.41%) was higher than in Shengeh (3.45%).

### 3.2 Differential expression gene analysis

The results of the differential expression gene (DEG) analysis showed that 2775 genes displayed statistically significant differential expression patterns at a 99% (FDR<0.01) (**Supplementary File 1**). Furthermore, the results indicated that the expression of 33% of the DEGs was up-regulated, while the expression of 67% of them was down-regulated. The 10 top DEGs with the highest differential expression levels are shown in **Table 1**. According to the obtained results in the up-regulated DEGs, the minimum Log-Fold change (LogFC) value is 2.13 and the maximum value is 10.20. Additionally, in the down-regulated DEGs, the minimum LogFC value is 2.13 and the maximum value is 12.62.

**Table 1:** The 10 top DEGs with highest differential expression levels in up and down-regulated genes.

Up-regulated			Down-regulated		
Gene ID	LogFC	FDR	Gene ID	LogFC	FDR
OE9A020293	10.20	5.02E-09	OE9A043283	-12.62	3.281E-15
OE9A102231	9.42	3.52438E-07	OE9A016722	-11.45	3.7357E-12
OE9A063930	9.29	6.69106E-07	OE9A011096	-11.34	7.5239E-12
OE9A006590	9.21	9.91751E-07	OE9A083183	-11.12	2.7308E-11
OE9A110764	9.11	1.56358E-06	OE9A055074	-11.12	2.7308E-11
OE9A088894	9.00	2.65746E-06	OE9A085340	-10.97	6.6778E-11
OE9A100739	8.83	6.27832E-06	OE9A001292	-10.946	7.1088E-11
OE9A010940	8.73	1.04085E-05	OE9A110790	-10.88	1.0473E-10
OE9A051863	8.61	1.77644E-05	OE9A050253	-10.63	4.4111E-10
OE9A067051	8.58	2.03889E-05	OE9A023437	-10.46	1.1856E-09

To investigate genes with varying expression levels (2775 genes), pathway enrichment analysis was conducted, and main pathways and key genes involved in fatty acid biosynthesis and oil quality were identified (**Table 2**) (**Supplementary Files 2**, **3**). In our previous study (Asadi et al., 2023), the effective pathways in the biosynthesis of fatty acids and their quality were identified. Examining the pathway enrichment results in the current study also validates that the identified pathways in the previous study are significant in two Iranian cultivars. Therefore, genes with differential expression in the galactose metabolism, glycolysis, fatty acid biosynthesis, biosynthesis of unsaturated fatty acids, fatty acid metabolism, glycerolipid metabolism, and cutin, suberin, and wax biosynthesis as pathways were further investigated. In the present study, glycolysis pathway has key roles for the supplying of acetyl-CoA. Also, the galactose metabolism pathway supplies carbon for the production of fatty acids, and fatty acid biosynthesis and biosynthesis of unsaturated fatty acids are key metabolic pathways for the biosynthesis of the carbon chain of fatty acids. In the assembly of fatty acids, the glycerolipid metabolism pathway was identified as an important metabolic pathway that can form complex lipids. The cutin, suberin, and wax biosynthesis pathway was identified as an important pathway in the production of by-products of fatty acid biosynthesis which can affect oil quality.

**Table 2:** Identified key genes in oil biosynthesis of the Mari and Shengeh.

DEG genes	Gene id	LogFC	FDR	Metabolic pathways
Raffinose synthase	OE9A043896	-5.67	1.26E-06	Galactose pathway
	OE9A046937	-5.41	9.99E-08	
	OE9A117492	-6.52	2.73E-11	
UDP-sugar pyrophosphorylase	OE9A052745	-3.41	6.19E-04	
UDP-glucose 4-epimerase enzyme	OE9A021881	2.36	3.75E-03	
Galactinol synthase	OE9A027274	-3.91	8.77E-05	
	OE9A068407	-2.48	6.13E-03	
	OE9A079169	5.16	3.40E-04	
	OE9A011682	3.78	1.25E-05	
Alpha-galactosidase	OE9A078785	-3.04	6.33E-04	
Phosphoglucomutase	OE9A037489	-2.58	2.41E-03	Glycolysis pathway
6-phosphofructokinase	OE9A084760	-7.50	1.55E-03	
Fructose-bisphosphate aldolase	OE9A026328	-2.26	7.53E-03	
Triose-phosphate isomerase	OE9A053216	-2.78	6.40E-03	
Pyruvate kinase	OE9A107193	-2.89	9.05E-03	
Pyruvate dehydrogenase	OE9A003942	-3.10	1.73E-04	
Pyruvate decarboxylase	OE9A088016	-4.95	7.56E-04	
	OE9A064738	-2.52	2.36E-03	
Acetyl-CoA synthetase	OE9A101525	-4.50	7.28E-06	
Beta-ketoacyl-[acp] synthase II	OE9A040462	-7.44	1.95E-03	Fatty acid biosynthesis and biosynthesis of unsaturated fatty acids
	OE9A104560	-3.14	3.50E-03	
Stearoyl-[acp] 9-desaturase	OE9A048475	2.26	6.13E-03	
Fatty acid desaturase 2	OE9A069627	-5.07	2.45E-05	
	OE9A098403	2.25	5.94E-03	
Long-chain acyl-CoA synthetase	OE9A097852	-5.80	1.22E-08	
	OE9A034515	-4.87	1.49E-07	
	OE9A095994	-2.18	9.68E-03	
CYP86A4	OE9A105123	-6.98	8.54E-03	
Peroxygenase	OE9A078287	4.01	2.75E-03	
Glycerol kinase	OE9A041104	-4.40	2.68E-06	Glycerolipid metabolism
Phosphatidate phosphatase	OE9A086145	-2.67	1.82E-03	
Diacylglycerol acyltransferase	OE9A108115	3.85	1.38E-05	
Sulfoquinovosyltransferase	OE9A075723	2.90	1.02E-03	

The negative LogFC values should be considered as up-regulated genes in Mari and down-regulated genes in Shengeh.

### 3.3 The synthesis of the carbon skeleton of fatty acids

Considering the importance and role of oligosaccharides as a source of carbon supply in the biosynthesis of fatty acids, the galactose pathway was investigated in Mari and Shengeh (**Supplementary File 4**). The results indicated that raffinose synthase (EC: 2.4.1.82) (OE9A043896, OE9A046937, OE9A117492) as a key enzyme for converting galactinol to raffinose, has a higher expression and probably increase the biosynthesis of raffinose in the Mari. In raffinose production, UDP-Galactose must be produced for the synthesis of galactinol as a precursor. The results indicated that UDP-Galactose is generated from two distinct metabolites in the Mari and Shengeh. In Mari, alpha-D-Galactose 1-phosphate is converted to UDP-Galactose by the up-regulation of UDP-sugar pyrophosphorylase (USP) (EC: 2.7.7.64) (OE9A052745) enzyme, whereas in Shengeh, UDP-glucose is converted to UDP-Galactose by the up-regulation of UDP-glucose 4-epimerase enzyme (UGE2) (EC: 5.1.3.2) (OE9A021881). Plants have two different routes to produce UDP-Galactose (Decker and Kleczkowski, 2019). The first path called the Leloir pathway (Leloir, 1951), was found in Shengeh and the second path, observed in Mari, involves a reaction catalyzed by USP (Dai et al., 2006). Based on the findings of current study, it can be assumed that Mari exhibits a more favorable pathway for UDP-Galactose production. In the next step, UDP-Galactose is converted into galactinol by the activity of the enzyme galactinol synthase (EC: 2.4.1.123), which is up-regulated in Mari (OE9A027274 and OE9A068407) and Shengeh (OE9A079169 and OE9A011682). Finally, the oligosaccharides are converted into simple sugars, they can be utilized in the synthesis of fatty acids and other compounds. The results indicate that the enzyme alpha-galactosidase (EC: 3.2.1.22) (OE9A078785), responsible for converting raffinose into simple sugars, is down-regulated in Shengeh. Our results show that Mari may have an effective mechanism relative to Shengeh for the production of oligosaccharides such as raffinose and simple sugars.

### 3.4 The generation of acetyl-CoA

The findings from the present research indicate that the glycolysis pathway plays a significant role in generating acetyl-CoA, a key precursor in the biosynthesis of fatty acids. The analysis of the glycolysis pathway indicates that the genes responsible for producing acetyl-CoA precursors, glyceraldehyde 3-phosphate (G3P), and pyruvate show increased expression in Mari, whereas there is no change in the expression of these genes in Shengeh (**Supplementary File 5**). Therefore, the results indicate that several enzymes were up-regulated in the Mari for the production of the G3P from alpha-D-glucose 1-phosphate. These enzymes include phosphoglucomutase (EC: 5.4.2.2) (OE9A037489), 6-phosphofructokinase (PFP-ALPHA1) (EC: 2.7.1.90) (OE9A084760), fructose-bisphosphate aldolase (EC: 4.1.2.13) (OE9A026328), and triose-phosphate isomerase (TIM) (EC: 5.3.1.1) (OE9A053216). The results of the present study demonstrate that the expression of TIM enzyme, which plays a vital role in the glycolysis pathway, increases at Jaén index four in the Mari. This enzyme facilitates the conversion of dihydroxyacetone phosphate to G3P and vice versa to maintain its level. Furthermore, fructose-bisphosphate aldolase enzyme was found to govern the production of crucial intermediate compounds, dihydroxyacetone phosphate and G3P, during oil biosynthesis. Another important up-regulated gene in Mari is pyruvate kinase (PKP4) (EC: 2.7.1.40) (OE9A107193), which converts phosphoenolpyruvate to pyruvate. The study’s findings also reveal that the second subunit of pyruvate dehydrogenase (pdhC or LTA2) (EC: 2.3.1.12) (OE9A003942) was up-regulated in Mari and involved in the conversion of pyruvate to acetyl-CoA. In contrast, the results from the Shengeh show down-regulation of pdhC (OE9A003942), indicating a lower activity of the pyruvate dehydrogenase complex at this stage. The findings reveal that at Jaén index four, maybe there is an alternative pathway for the conversion of pyruvate to acetyl-CoA in the Mari. In this pathway, pyruvate is first converted to acetaldehyde by up-regulation of pyruvate decarboxylase (PDC2) (EC: 4.1.1.1), and then acetaldehyde can be further converted to acetate. Finally, the enzyme acetyl-CoA synthetase (ACS) (EC: 6.2.1.1) (OE9A101525) is up-regulated and can convert acetate to acetyl-CoA in the Mari. This indicates that Mari can have a compensatory mechanism in place for the production of acetyl-CoA at Jaén index four. On the other hand, PDC2 (EC: 4.1.1.1) (OE9A088016 and OE9A064738) is down-regulated in the Shengeh, suggesting that the compensatory mechanism is not active in this cultivar.

### 3.5 Biosynthesis of fatty acids

Increasing the number of carbons in the fatty acid carbon chain and producing stearoyl-ACP (C18:0) is a crucial step in the biosynthesis of fatty acids in olive. This process involves elongating the carbon chain to form stearoyl-ACP from palmitoyl-ACP (C16:0) by FabF (EC: 2.3.1.179). The results of the current study show that the expression of the FabF enzyme is up-regulated in the Mari (OE9A040462 and OE9A104560) which can lead to an increase in the carbon chain length of fatty acids from 16 to 18 carbons (**Supplementary File 6**). This process can transform palmitic acid (C16:0) into stearic acid (C18:0), enhancing oil quality by decreasing palmitic acid levels and boosting stearic acid content, which serves as a precursor to oleic acid. The GC results also demonstrate that the total amount of 18 carbon fatty acids (86.04%) in the Mari is higher compared to the 16 carbon fatty acids (81.13%). Furthermore, the GC results reveal that the total amount of 18 carbon fatty acids in the Mari (86.04%) is greater than in the Shengeh (77.01%) at Jaén index four, which can be assumed to the higher activity of the FabF enzyme in the Mari.

Desaturation is the key step in oil biosynthesis and the results show that in Shengeh, the SAD enzyme (EC: 1.14.192) (OE9A048475) is up-regulated at Jaén index four, leading to the production of fatty acids with double bonds (**Supplementary File 6**). Shengeh’s pathway analysis shows a decline in FabF enzyme expression, while the SAD enzyme expression rises, leading to the formation of fatty acids with a 16-carbon chain and a double bond, particularly palmitoleic acid. The GC results also reveal that the level of palmitoleic acid in Shengeh (2.37%) is higher than in Mari (0.94%). Fatty acid desaturases (FAD) enzymes which convert oleic acid to linoleic acid, is another key enzyme in desaturation step. In the present study, two different genes for the fatty acid desaturase 2 (FAD2) (EC: 1.14.19.6 and EC: 1.14.19.22) enzyme were identified in each of the studied cultivars. In Mari, OE9A069627 was identified, which was up-regulated and OE9A098403 was identified, which was up-regulated in Shengeh. The results show that in Mari, the expression of the OE9A098403 is down-regulated compared to Shengeh. This down-regulation can lead to a decrease in linoleic acid production and the maintenance of oleic acid levels at this stage of growth in Mari. Furthermore, the results of current study show that the expression of the SAD enzyme is up-regulated (OE9A048475) in Shengeh which can increase the production of oleic acid. However, the simultaneous up-regulation of FAD2 with the SAD enzyme converts oleic acid to linoleic acid. Our GC results also indicate that the level of linoleic acid in Shengeh (26.69%) is higher than in Mari (4.76%), and Mari can have a higher level of oleic acid due to the lower activity of FAD2. Therefore, the higher level of oleic acid in Mari can be supposed to be the decrease in FAD2 expression and the lack of synchronization of its activity with the SAD enzyme.

One of the important enzymes in the fatty acid synthesis and catabolism is long-chain acyl-CoA synthetase (LACS) (EC: 6.2.1.3) which adds a CoA group to the palmitic acid and finally forming long-chain acyl-CoA (Liu et al., 2021). The results showed that the expression of the LACS enzyme (OE9A097852, OE9A034515, and OE9A095994) was up-regulated in Mari at Jaén index four. Utilizing palmitic acid in the production of long-chain fatty acids can effectively improve oil quality. The GC results also indicate that the amount of palmitic acid in Mari (12.87%) is lower compared to Shengeh (20.16%), suggesting that the increased expression of the LACS enzyme probably potentially be considered as an effective factor in reducing the quantity of this fatty acid. Furthermore, the results indicate an increase in the expression of the CYP86A4 (OE9A105123) enzyme in Mari which converts palmitic acid to Juniperic acid, a crucial monomer of cutin in plant cuticles (**Supplementary File 7**). In contrast, the results reveal that in the Shengeh at this growth stage, the peroxygenase enzyme (EC: 1.11.2.3) (OE9A078287) up-regulated and utilizes oleic acid to produce compounds like waxes. Therefore, the enzymes identified in the decomposition of palmitic acid may be effective in increasing the quality of olive oil.

### 3.6 Assembly of fatty acid to form triacylglycerols

After the production of fatty acids, triacylglycerols (TAG) is made up by four reactions in the glycerolipid metabolism pathway. Our findings indicated that the levels of glycerol kinase (GLPK) (EC: 2.7.1.30) (OE9A041104) were reduced in the Shengeh, leading to the inhibition of glycerol conversion to G3P (**Supplementary File 8**). This decrease hinders the initiation of key processes essential for diacylglycerols (DAG) production. For the direct synthesis of DAG, which serve as the major precursor for TAG, the enzyme phosphatidate phosphatase (PAH2) (EC: 3.1.3.4) converts phosphatidic acid to DAG. The findings of this study indicate that the activity of PAH2 enzyme (OE9A086145) is down-regulated in Shengeh. As a result, it is probable that phosphatidic acid and DAG are the main precursors for TAG production. Two pathways exist for the production of TAG from DAG. The first pathway relies on the presence of fatty acids, where DAG is converted to TAG by the enzyme diacylglycerol acyltransferase (DGAT). The second pathway operates independently of fatty acids, with DAG being transformed into TAG by the enzyme phospholipid:diacylglycerol acyltransferase (PDAT). The findings of current study reveal that DGAT (EC: 2.3.1.20) (OE9A108115) is up-regulated in Shengeh. Furthermore, the results indicate that the expression of the enzyme sulfoquinovosyltransferase (SQD2) (EC: 2.4.1.-) (OE9A075723) is up-regulated in Shengeh, suggesting that DAG can be converted into other glycerolipids.

### 3.7 Transcription factor identification and their interactions

In order to identify the effective transcription factors (TF) in oil quality, the protein sequence of DEGs was compared in the PlantTFDB website, and 78 TFs were identified (**Supplementary File 9**). All identified TFs were significant (p-value<0.05) and the family associated with each TF was also determined. Examining the frequency of the family of TFs showed that bHLH (11.54%) and WRKY (10.26%) families have the highest frequency (**Supplementary File 10**). One important TF involved in oil biosynthesis is WRINKLED1 (WRI1), which is a member of the AP2/EREBP family of TFs. The WRI1 gene encodes a TF belonging to the APETALA2 (AP2) family (Kong et al., 2020). The current research has recognized the AP2 family (2.56%) as one of significant group of TFs in olive.

**Figure 2:**
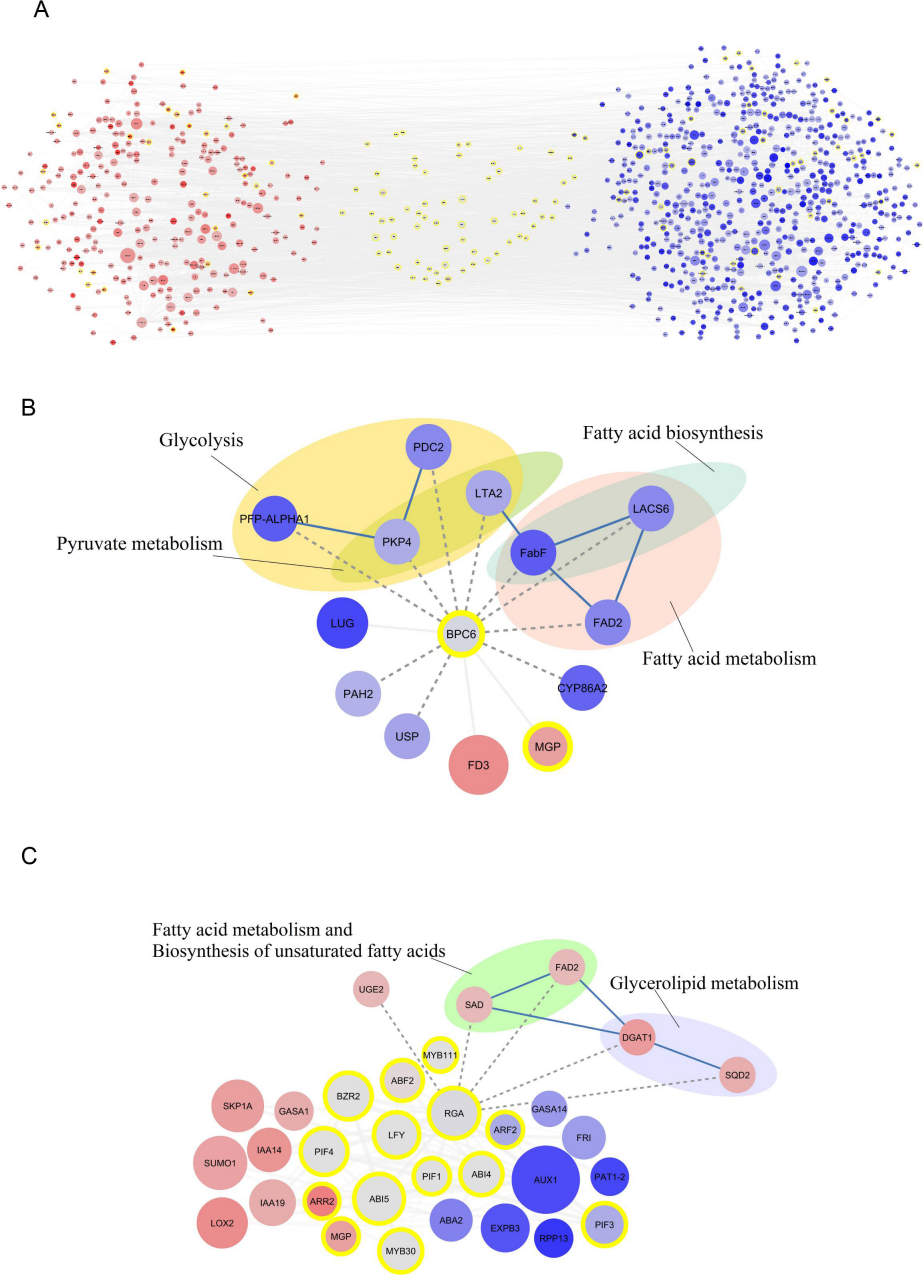
Protein-protein interaction network of differentially expressedn genes and identified transcription factors **(A)**. The interaction of BPC6 **(B)** and RGA **(C)** and their target genes. The up-regulated and down-regulated genes are shown in red and blue color, respectively. The intensity of the color also indicates the level of gene expression, as darker colors indicate the highest expression. The transcription factors are marked with yellow circles and also transcription factor enrichment’s results are marked with dashed lines. The metabolic pathways related to the identified target genes for each transcription factor are also shown. (PFP-ALPHA1, 6-phosphofructokinase; PDC2, pyruvate decarboxylase; PKP4, pyruvate kinase; LTA2, second subunit of pyruvate dehydrogenase; FabF, beta-ketoacyl-[acp] synthase II; FAD2, fatty acid desaturase 2; PAH2, phosphatidate phosphatase; USP, UDP-sugar pyrophosphorylase; SAD, stearoyl [acyl-carrier-protein] 9-desaturase; UGE2, UDP-glucose 4-epimerase enzyme; DGAT1, Diacylglycerol acyltransferase; SQD2, sulfoquinovosyltransferase).

The study investigated the upstream regions of both down-regulated and up-regulated genes separately, leading to the identification of associated TF regions. The study revealed that 95 significant regions linked to TFs were found upstream of the down-regulated key genes, which contained 2 to 10 target genes (**Supplementary File 11**). The BPC6 TF showed the most target genes, with 10 target genes found upstream of the main down-regulated genes (**Figure 2B**). In contrast, the expression of these TFs was assessed, showing a reduction in BPC6 expression in the Shengeh. Additionally, the current study demonstrated that seven significant regions linked to TFs were found upstream of the up-regulated key genes, which contained 2 to 5 target genes (**Supplementary File 12**). The findings revealed that the RGA boasts the highest number of target genes compared to other TFs, harboring five target genes upstream of the key up-regulated genes (**Figure 2C**). Analysis of the RNA-seq data unveiled a decrease in the expression of this TF in the Shengeh, prompting an increase in the expression of the target genes. Protein–protein interactions of DEGs and identified TFs were drawn and the network included 764 nodes and 1951 edges (**Figure 2A**).

#### 3.7.1 The interaction of BPC6 and target genes

**Figure 3:**
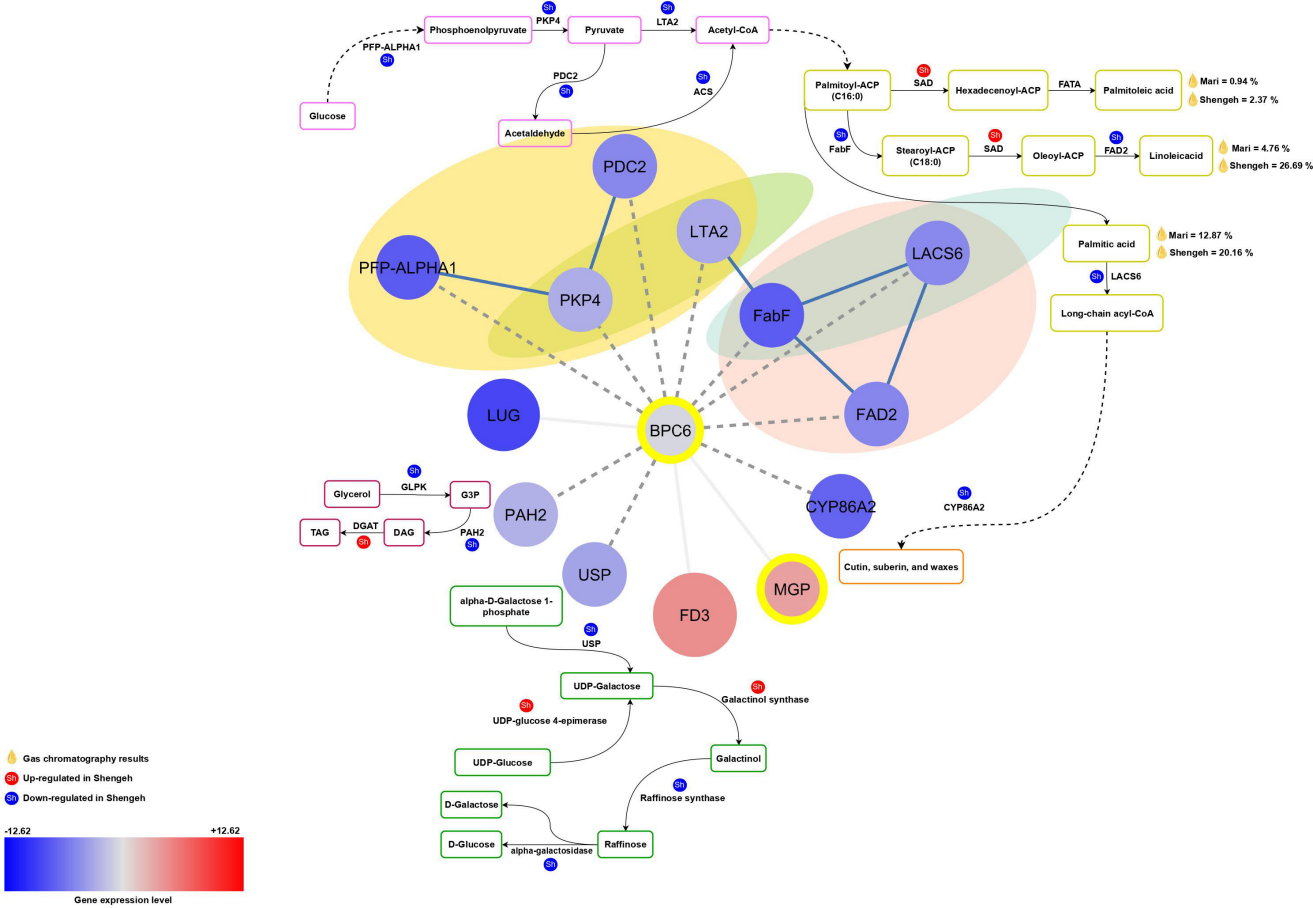
The relationship between BPC6 and its target genes in various essential pathways. The red color depicts up-regulated genes and the blue color represents down-regulated genes. The intensity of the color also indicates the level of gene expression. The transcription factors are marked with yellow circles and also transcription factor enrichment’s results are marked with dashed lines. (PFP-ALPHA1, 6-phosphofructokinase; PDC2, pyruvate decarboxylase; PKP4, pyruvate kinase; LTA2, second subunit of pyruvate dehydrogenase; FabF, beta-ketoacyl-[acp] synthase II; FAD2, fatty acid desaturase 2; PAH2, phosphatidate phosphatase; USP, UDP-sugar pyrophosphorylase; SAD, stearoyl [acyl-carrier-protein] 9-desaturase; ACS, acetyl-CoA synthetase (ACS); G3P, glyceraldehyde 3-phosphate; DAG, Diacylglycerols; TAG, Triacylglycerols).

The BPC6 (OE9A013666; AT5G42520) TF regulates important genes like PFP-ALPHA1, PKP4, PDC2, LTA2, FabF, FAD2, LACS6, CYP86A2, and USP (**Figure 3**). A decrease in BPC6 expression may lead to reduced expression of these genes, impacting various pathways. In the galactose pathway, lower BPC6 expression reduces USP enzyme expression, affecting raffinose production. Reduced BPC6 expression in glycolysis can result in lower PFP-ALPHA1 and PKP4 expression and may be impacting pyruvate production. Probably, decreased LAT2 enzyme expression due to reduced BPC6 affects acetyl-CoA production and fatty acid biosynthesis. Lower PDC2 expression may impact acetyl-CoA production. The FabF enzyme expression decreases and can change 16/18 fatty acid ratios may lead to an increase in the production of palmitic acid and palmitoleic acid in the Shengeh and reduced oil quality. Reduced LACS6 expression can affect long-chain fatty acid production and decreased CYP86A2 expression may reduce cutin, wax, and suberin production.

#### 3.7.2 The interaction of RGA and target genes

**Figure 4:**
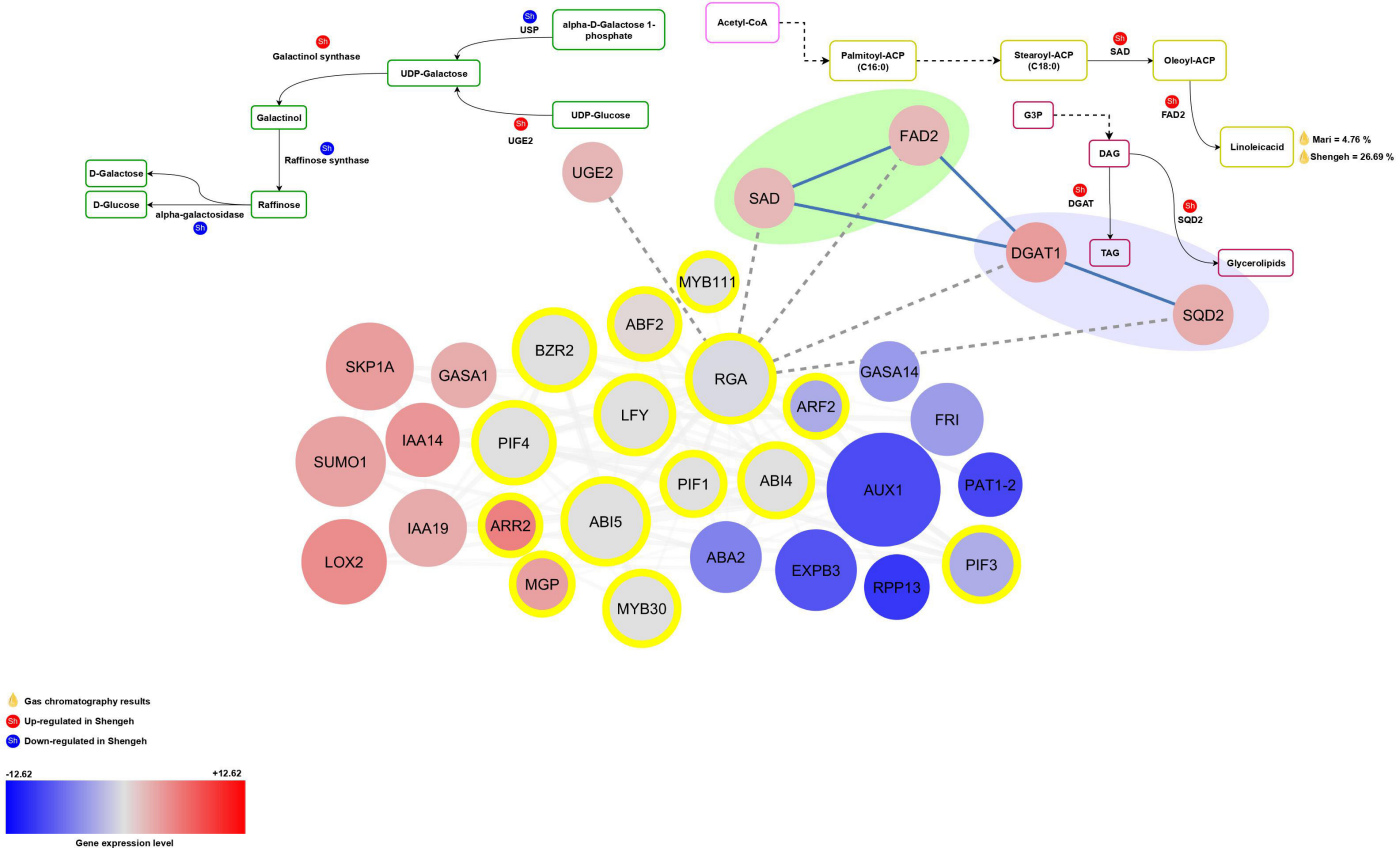
The relationship between RGA and its target genes in various essential pathways. The red color depicts up-regulated genes and the blue color shows down-regulated genes. The intensity of the color also indicates the level of gene expression. The transcription factors are marked with yellow circles and also transcription factor enrichment’s results are marked with dashed lines. (UGE2, UDP-glucose 4-epimerase enzyme; FAD2, fatty acid desaturase 2; USP, UDP-sugar pyrophosphorylase; SAD, stearoyl [acyl-carrier-protein] 9-desaturase; DGAT1, Diacylglycerol acyltransferase; SQD2, sulfoquinovosyltransferase; G3P, glyceraldehyde 3-phosphate; DAG, Diacylglycerols; TAG, Triacylglycerols).

The RGA (OE9A032653 and OE9A011576; AT2G01570) is involved in controlling the expression of UGE2, SAD, FAD2, DGAT1, and SQD2 genes. Reduction in RGA expression may lead to an up regulation of these genes (**Figure 4**). The results show that the presence of RGA upstream of the UGE2 gene probably increase the activity of this enzyme in the Shengeh and increase the activity of this pathway for the production of UDP-Galactose, so that probably the production of raffinose increase. According to the obtained results, the greatest role of the RGA could be related to the production of unsaturated fatty acids in the fatty acid biosynthesis pathway. The RGA is located upstream of two key genes SAD and FAD2, which are involved in the production of unsaturated fatty acids. The results show that the decrease in the expression of the RGA may increase the expression of SAD and FAD2 genes in the Shengeh toward the production of linoleic acid. According to the obtained results, it can be assumed that the negative regulation of the key genes SAD and FAD2 by the RGA can be one of the reasons for the decrease in oil quality in the Shengeh at Jaén index four. Also, the results show that in the Shengeh, the decrease in the expression of the RGA increases the expression of the DGAT1 enzyme, which plays a role in TAG production. On the other hand, decreasing the expression of the RGA can play a role in increasing the expression of the SQD2 enzyme in this pathway, and this enzyme can increase the production of glycerolipids.

## 4 Discussion

In the present study, we compared two Iranian extreme olive cultivars, Mari and Shengeh, for expression of key genes in the biosynthesis of fatty acids and oil quality at Jaén index four. The results of GC analysis showed that Mari has a better quality than Shengeh due to high oleic acid and low palmitic acid and linoleic acid. In a research conducted by Parvini et al. (2015), it was stated that the percentage of oleic acid in mesocarp of Mari was much higher than Shengeh. We also evaluated the O/L ratio as a crucial factor in assessing the nutraceutical potential and high stability of the examined cultivars. In a study conducted by Parvini et al. (2014), on Mari and Shengeh cultivars at 45 to 200 DAF at the Tarem Olive Research Station, it was found that the average O/L ratio in Mari is significantly high (27.79%) compared to Shengeh (3.15%), a result that aligns with the findings of the current study.

Sugars are one of the main elements that are used as precursors of oil biosynthesis in olive fruits (Salas et al., 2013). Leaf photosynthesis is the main and fruit photosynthesis is a secondary important source of carbohydrate production for mesocarp growth and the production of the carbon skeleton of fatty acids in olive (Conde et al., 2008; Salas et al., 2013). In addition to leaf and fruit photosynthesis, the ability to produce oligosaccharides as a separate reaction is a special feature in plants of the *Oleaceae* family and some other plant families, such as *Apiaceae* (Sánchez and Harwood, 2002; Salas et al., 2013). In the research conducted by de Koning et al. (2021) indicate that raffinose synthase is expressed from the middle of seed maturity stage until the end of maturation, playing a crucial role in the accumulation of raffinose in soybean and common bean seeds. A study conducted by Valentine et al. (2017), demonstrated that silencing raffinose synthase gene in *Glycine max* resulted in a significant decrease in the content of raffinose and stachyose in the seeds. Another crucial enzyme involved in raffinose production is galactinol synthase, and its expression varies between the two cultivars examined in the current study. According to a study by Le et al. (2020), knocking the genes responsible for galactinol synthase in soybeans results in reduced raffinose production, causing alterations in carbohydrate levels. Therefore, it can be assumed that the expression of enzymes involved in raffinose production and conversion to simple sugars is up-regulated in Mari at Jaén index four. This compensatory mechanism is properly active in providing carbon for fatty acid biosynthesis in this cultivar. Furthermore, towards the end of the growth stage, when fruit photosynthesis decreases as a result of darkening color, Mari can exhibits an efficient carbon supply mechanism.

The glycolysis is stable and rapid way in acetyl-CoA production rather than pentose phosphate (Asadi et al., 2023). Additionally, in olives, the glycolysis pathway and the production of acetyl-CoA predominantly occur in plastids rather than mitochondria and olives have a preference for synthesizing acetyl-CoA and fatty acids within one cellular organelles (plastids) (Harwood and Aparicio, 2000). In general, it can be supposed that the expression of genes in the glycolysis pathway is significantly higher in the Mari compared to Shengeh. A study on Arabidopsis showed that mutants lacking phosphoglucomutase, a key enzyme in G3P production, had a 40% decrease in oil content compared to the wild type. Inhibiting the conversion of Glucose 1-phosphate to Glucose 6-phosphate in these mutants limited carbon flow to fatty acid synthesis, leading to the reduction in oil content (Periappuram et al., 2000). In other hand, by increasing the activity of phosphoglucomutase and TIM in Mari, the conversion of G3P and dihydroxyacetone phosphate is facilitated, enabling a continuous flow of metabolites in glycolysis, leading to the production of pyruvate. Another enzyme that plays a key role in generating pyruvate and acetyl-CoA is fructose-bisphosphate aldolase, which is up-regulated in Mari. Previous research has highlighted its relationship with the expression levels of the SAD enzyme (Manan et al., 2017b). In a study by Guerin et al. (2016) on the gene co-expression network of the oil biosynthesis pathway in palm trees, it was discovered that the enzymes 6-phosphofructokinase and PKP4, which are important for pyruvate and acetyl-CoA biosynthesis, directly interact with genes involved in fatty acid biosynthesis. The results of the present study show that the accumulation of pyruvate in order to produce acetyl-CoA continues up to Jaén index four in Mari. Subsequently, pyruvate is converted to acetyl-CoA by the activity of pyruvate dehydrogenase complex (Lei et al., 2022). According to results of Asadi et al. (2023), the highest rate of pyruvate accumulation is observed at 90 DAF, and only pdhC is active at 130 DAF and beyond. In Arabidopsis, the analysis of pdhC and acetyl-CoA synthetase, essential enzymes in Acetyl-CoA production, showed that acetyl-CoA generated from acetate via the acetyl-CoA synthetase pathway is not a major source for fatty acid biosynthesis. Rather, the plastidic pdhC reaction plays a critical function in this process (Lin and Oliver, 2008). Study of this matter in Mari revealed that the activity of pdhC in producing acetyl-CoA is enhanced and plays a more crucial role compared to the compensatory mechanism involving acetate. In contrast, examination of the alternative pathway for acetyl-CoA production from acetate in Shengeh indicated a decrease in gene expression along this pathway. In a study by Guerin et al. (2016) on the gene co-expression network of palm oil biosynthesis, it was discovered that enzymes such as aldehyde dehydrogenase and acetyl-CoA synthetase, found in the compensatory mechanism, indirectly influenced the biosynthesis of fatty acids.

The FabF enzyme plays a crucial role in determining the ratio of C16/C18 fatty acids and directly affects the degree of unsaturation of the oil, subsequently impacting the quality of the oil (Špika et al., 2021). Based on the GC results and the up-regulation of the FabF enzyme in Mari, the higher ratio of 18-carbon to 16-carbon fatty acids in this cultivar may be attributed to the function of the FabF enzyme. In a research study comparing the fatty acid composition at five different growth stages of olives, it was observed that the FabF enzyme expression peaked at 80 DAF, can lead to a higher proportion of 18-carbon fatty acids compared to 16-carbon fatty acids (Liu et al., 2021). Research conducted on oil palm and canola has revealed a negative correlation between the FabF enzyme and C16:0 fatty acids, while simultaneously increasing the content of C18:0 (Gupta et al., 2012; Guerin et al., 2016). The mutation of FabF in *Escherichia coli* led to a shift in the fatty acid distribution, with a significant increase in palmitic acid and palmitoleic acid levels, making up over 70% of the total fatty acids. Additionally, when the FabF gene from *Arabidopsis thaliana* was cloned, there was a notable increase in the levels of C18 fatty acids (Kassab et al., 2019). The desaturation of 18-carbon fatty acids like stearoyl-ACP can enhance oleic acid production and enhance the quality of oil. In the research conducted by Parvini et al. (2015), it is also stated that the highest activity of the SAD enzyme in the Mari is observed at 120 DAF and decreases at 150 DAF, which is consistent with the results of the present study. The results of the meta-analysis conducted on olive indicate that the activity of the SAD enzyme reaches its peak at 90 DAF and subsequently decreases after 130 DAF (Asadi et al., 2023). Based on this finding, it can be inferred that the activity of the SAD enzyme in the Mari, which contains higher levels of oleic acid, likely increases prior to Jaén index four. The results of previous studies show that the maximum expression of the FabF in 80 DAF and the maximum activity of the SAD before 120 DAF can be an effective solution to increase the level of oleic acid in cultivars such as Mari that have high oleic acid. Preventing the conversion of oleic acid to linoleic acid by reducing the expression of FAD2 is another method that helps to maintain the level of oleic acid. In a research conducted by Liu et al. (2021), the transcript OE6A098403, up-regulated in Shengeh, exhibited elevated expression levels compared to other FADs, potentially leading to an increase in linoleic acid content in Shengeh. Also, the results of Sirangelo et al. (2023) show that FAD2 is down-regulated in the ripening stages in genotypes with high oleic acid. Based on the obtained results, the simultaneous activity of the FAD2 and SAD enzymes can be considered as one of the criteria for reducing oil quality at Jaén index four. The findings from the research conducted by Sirangelo et al. (2023) indicate that during the ripening stage of fruit in genotypes with high oleic acid and low linoleic acid (such as Mari), there is a down-regulation of the FAD2 gene and an up-regulation of the SAD gene. In a study by Unver et al. (2017), it was noted that the decrease in FAD2 expression and increase in SAD expression correlated with the accumulation of higher levels of C18:1 in olive. On the other hand, in the research conducted by Hu et al. (2023), FAD2 showed significantly higher expression compared to SAD, supporting the conversion of C18:1 and correlating with the increased presence of C18:2 in genotypes with high linoleic acid. The integrated analysis of genes involved in the fatty acid biosynthesis demonstrated that the optimal coordination between high-expression SAD and low-expression FAD enzymes facilitated the significant accumulation of oleic acid in cultivars such as Mari.

Conversely, the AP2/ERF superfamily stands out as one of the most extensive collections of plant-specific TFs. Furthermore, the AP2/ERF superfamily can be categorized into three subfamilies: AP2, RAV, and ERF (Yamada et al., 2020). In our study, we discovered all subfamilies - AP2, RAV, and ERF - where the ERF (7.69%) exhibited a higher frequency compared to the RAV (1.28%) and AP2 (2.56%) families. Previous research on oil seeds has highlighted the significance of MYB, WRKY, bZIP, Dof, and Hox TFs in oil biosynthesis (Yang et al., 2022); in our investigation, we also identified MYB, WRKY, bZIP, and Dof as influential factors. One of the important TFs in fatty acid biosynthesis is the WRI1, which controls many genes of the fatty acid biosynthesis pathway. The upstream sequences of the genes controlled by the WRI1 have high similarity to the sequences of the BBR/BPC family TFs. Therefore, the upstream sequence of genes regulated by WRI1 is known as AW-Box, and this region probably has a high similarity with the BPC6 sequence upstream of identified target genes (Kong et al., 2020; Kuczynski et al., 2022). The RNA-seq results of the present study show that the expression of the gene encoding the TF WRI1 (OE9A064028) is down-regulated in the Shengeh and can also reduce the expression of the target genes controlled by this TF. Therefore, the key and effective genes in the biosynthesis of fatty acids identified in the present study in Mari and Shengeh can be controlled by BPC6 and WRI1. In a research conducted by Yan et al. (2021), the role of gibberellin in the biosynthesis of fatty acids in *Brassica napus* was investigated and four RGA encoding genes were identified that negatively controlled the gibberellin signaling pathway. In the mentioned research, it has been stated that the creation of mutations in all four identified genes causes an increase in the amount of seed oil content at the end of the seed development. On the other hand, in the study of Yan et al. (2021), the interaction of the RGA with another TF such as LEC at the beginning of the seed development causes the amount of seed oil to decrease, and at the end of the growing season, the activity of the FAD2 enzyme decreases and the amount of linoleic acid decreases. Therefore, it can be said that in rapeseed LEC2 TF suppresses the FAD2 enzyme and the RGA may reduce this suppressive effect.

## 5 Conclusion

The present study compared two Iranian extreme olive cultivars, Mari and Shengeh, for the expression of key genes in the biosynthesis of fatty acids and oil quality at Jaén index four. The results indicated that Mari exhibited superior quality compared to Shengeh, characterized by higher oleic acid and lower palmitic acid and linoleic acid levels. The differential gene expression analysis identified 2775 genes with statistically significant expression patterns, with 33% of DEGs up-regulated and 67% down-regulated. Pathway enrichment analysis revealed key genes involved in fatty acid biosynthesis and oil quality pathways, highlighting the importance of pathways such as glycolysis, galactose metabolism, fatty acid biosynthesis, and glycerolipid metabolism in the biosynthesis of fatty acids. The study highlighted the importance of oligosaccharides as a source of carbon supply for oil biosynthesis in olive fruits. Enzymes involved in raffinose production and conversion to simple sugars were found to be up-regulated in Mari, indicating an efficient carbon supply mechanism for fatty acid biosynthesis in this cultivar. Moreover, the study revealed that Mari exhibited higher expression of genes in the glycolysis pathway compared to Shengeh, leading to enhanced acetyl-CoA production and fatty acid biosynthesis. The up-regulation of enzymes such as phosphoglucomutase, 6-phosphofructokinase, fructose-bisphosphate aldolase, triose-phosphate isomerase, and pyruvate dehydrogenase in Mari contributed to increased acetyl-CoA production. Furthermore, the research emphasized the significance of the FabF enzyme in influencing the ratio of C16/C18 fatty acids and its impact on oil quality. The up-regulation of FabF in Mari resulted in a higher proportion of 18-carbon fatty acids, contributing to the enhanced quality of the oil. The coordinated expression of SAD and FAD2 enzymes in Mari facilitated the accumulation of oleic acid and maintained oil quality. A different approach observed in Mari to maintain oleic acid levels involves using alternative fatty acids like palmitic acid to generate different by-products. The findings show an elevation in the expression of the CYP86A4 enzyme in Mari, may be responsible for converting palmitic acid into Juniperic acid and in Shengeh at the same growth stage, the peroxygenase enzyme is up-regulated and can employs oleic acid to create waxes.

## Data Availability

The datasets presented in this study can be found in online repositories. The names of the repository/repositories and accession number(s) can be found in the article/**Supplementary Material**. The raw RNA-seq datasets for this study can be found in the National Center for Biotechnology Information (NCBI) Sequence Read Archive (SRA). The transcriptome project has been deposited under the accession number PRJNA1111836 (https://www.ncbi.nlm.nih.gov/sra/PRJNA1111836).
