# Supplementary material for: Unraveling the genetic basis of oil quality in olives: a comparative transcriptome analysis
Source: Front Plant Sci. 2024 Oct 1;15:1467102. doi: 10.3389/fpls.2024.1467102 (PMC11473408; doi:10.3389/fpls.2024.1467102)
Supplement: Supplementary file 1 [file DataSheet1.zip › Data Sheet 1/Supplementary file 4.docx]

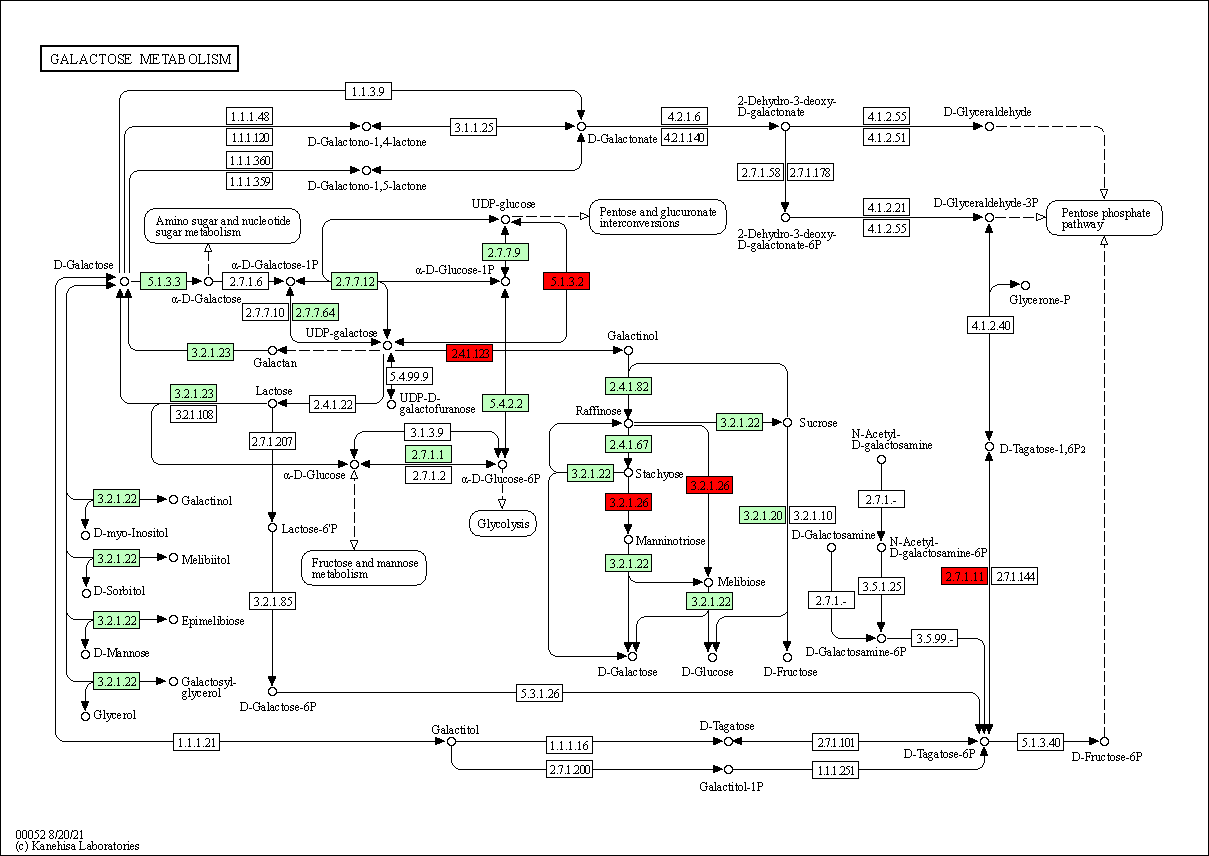


Figure S1. Up-regulated identified genes of the galactose metabolism pathway in the biosynthesis of raffinose family oligosaccharides


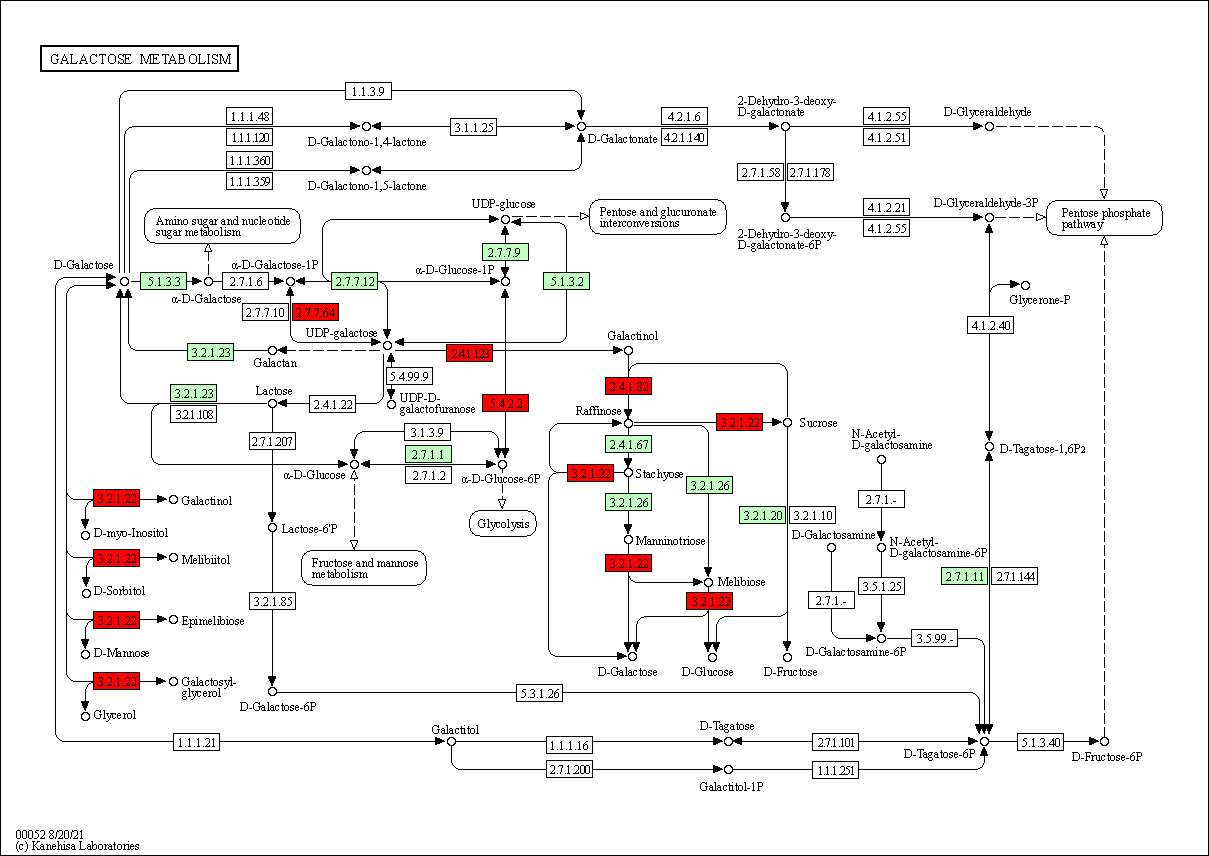


Figure S2. Down-regulated identified genes of the galactose metabolism pathway in the biosynthesis of raffinose family oligosaccharides
