# Supplementary material for: Unraveling the genetic basis of oil quality in olives: a comparative transcriptome analysis
Source: Front Plant Sci. 2024 Oct 1;15:1467102. doi: 10.3389/fpls.2024.1467102 (PMC11473408; doi:10.3389/fpls.2024.1467102)
Supplement: Supplementary file 1 [file DataSheet1.zip › Data Sheet 1/Supplementary file 7.docx]

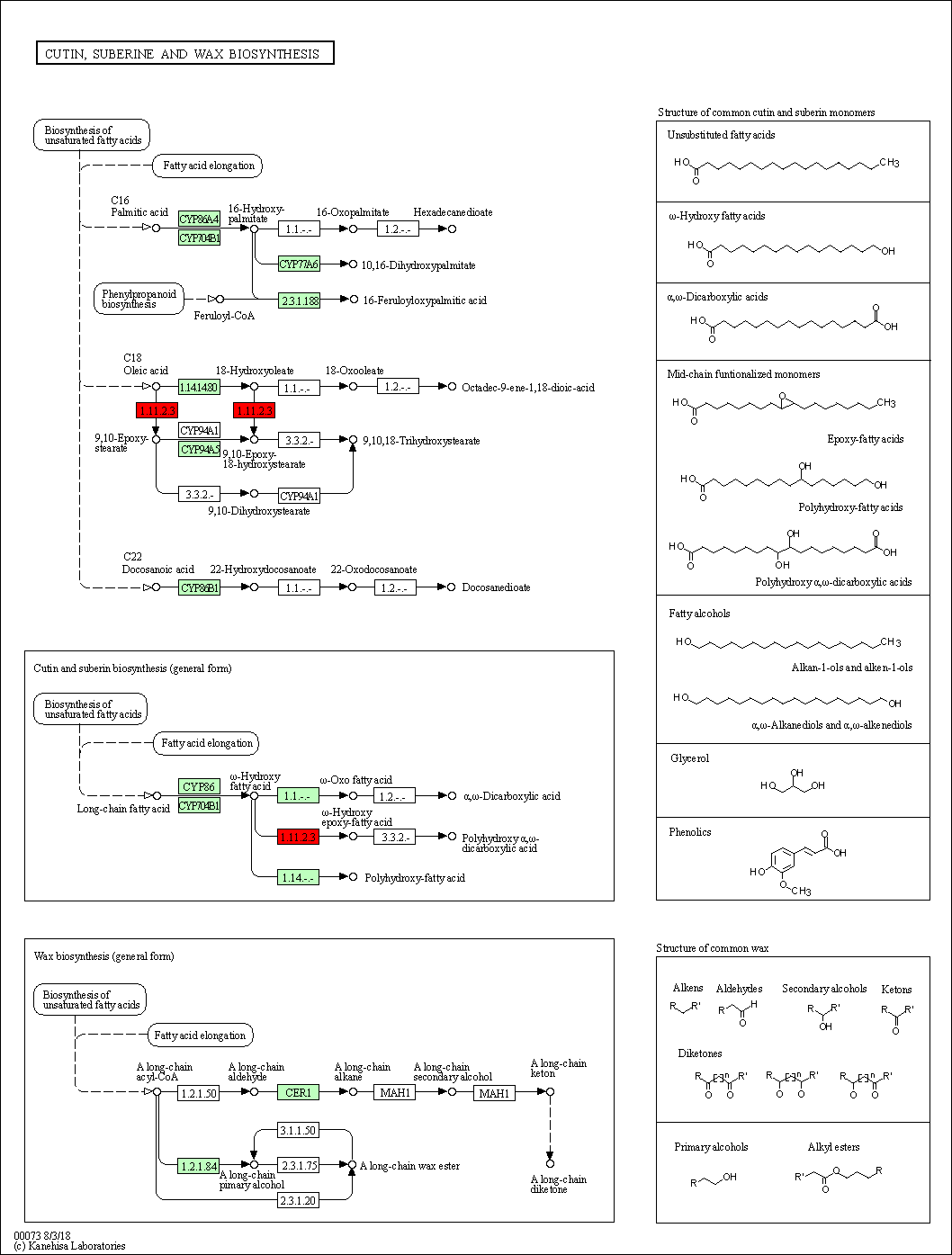


Figure S7. Up- regulated identified genes in the cutin, suberine, and wax biosynthesis pathway


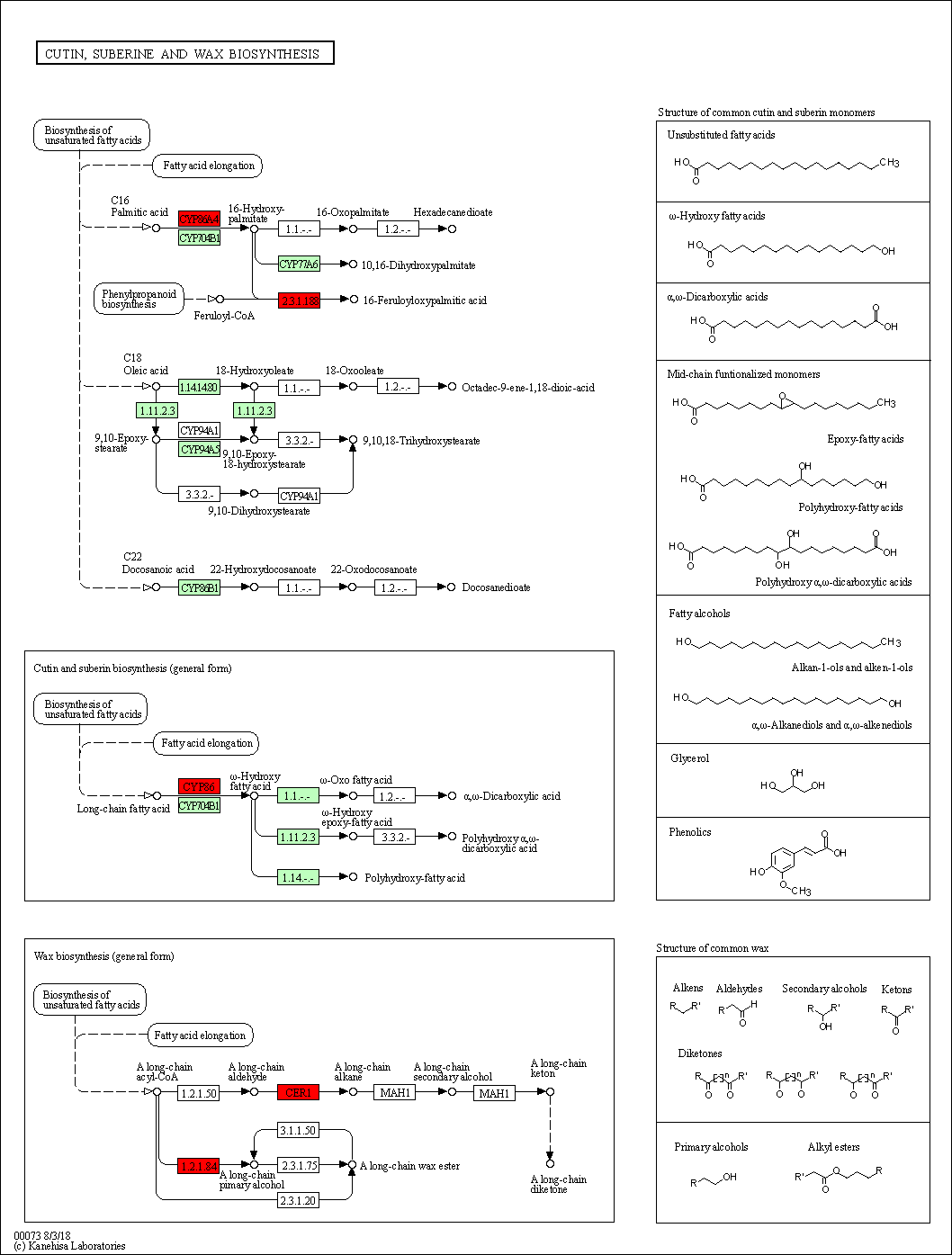


Figure S8. Down- regulated identified genes in the cutin, suberine, and wax biosynthesis pathway
