# Supplementary figures and images for: Unraveling the genetic basis of oil quality in olives: a comparative transcriptome analysis
Source: Front Plant Sci. 2024 Oct 1;15:1467102. doi: 10.3389/fpls.2024.1467102 (PMC11473408; doi:10.3389/fpls.2024.1467102)

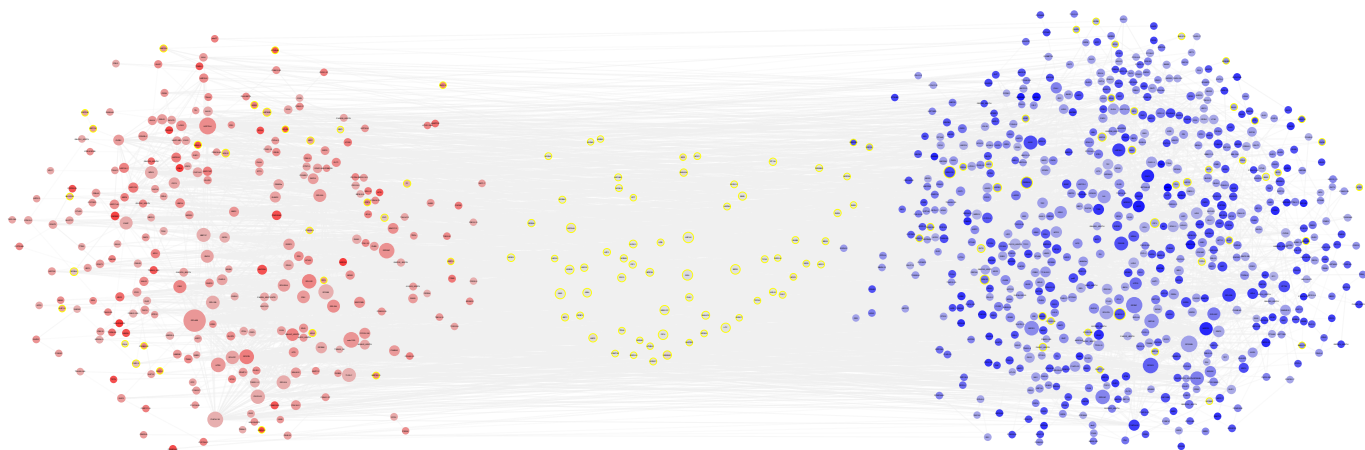

Supplement: Supplementary file 1 [file DataSheet1.zip › Data Sheet 1/Figure 2A.PDF]
